# Two ratiometric fluorescent sensors originating from functionalized R6G@UiO-66s for selective determination of formaldehyde and amine compounds†

**DOI:** 10.1039/d5ra01251a

**Published:** 2025-05-06

**Authors:** Wanqiao Bai, Zhuojun Zhao, Ting Zhang, Hongmei Chai, Loujun Gao

**Affiliations:** a Shaanxi Key Laboratory of Chemical Reaction Engineering, College of Chemistry and Chemical Engineering, Yan'an University Yan'an 716000 P. R. China glj@yau.edu.cn baiwanqiao@yau.edu.cn +86 911 2650317

## Abstract

Residual small amounts of harmful substances in food or medicine are potential threats to human health. In this work, amino-functionalized UiO-66 was firstly prepared, namely UiO-66-(a), then it was further treated with phosgene to obtain UiO-66-(b) with abundant carboxyl groups. By doping, the fluorescent Rhodamine 6G (R6G) was incorporated into the structures of the two functional UiO-66s to obtain R6G@UiO-66-(a) and R6G@UiO-66-(b), respectively. These two materials can both emit fluorescence based on UiO-66s and R6G, therefore, were employed as fluorescent probes to construct two ratiometric fluorescent sensors to detect formaldehyde and amine compounds, respectively. Based on the aldehyde-amine condensation reaction between –NH_2_ and –CHO and the specific condensation reaction between –COOH and –NH_2_, formaldehyde molecules and amine compounds can react with these two materials, respectively. Causing a change in the relative fluorescence intensity of functionalized MOFs, resulting in selective detection of formaldehyde and amine compounds with the detection limit of 0.058 μM and 0.0017 μM (ethylenediamine), respectively. These two ratiometric fluorescent probes were successfully applied for quantitative detection of formaldehyde in beer and ethylenediamine in anti-inflammatory agents, demonstrating great practical potential for residual hazardous substance monitoring in food or medicine.

## Abstract 2

Two ratiometric fluorescence sensing systems based R6G@UiO-66-(a) and R6G@UiO-66-(b) were designed to detect formaldehyde and ethylenediamine with selective and reliable result.

## Graphical abstract

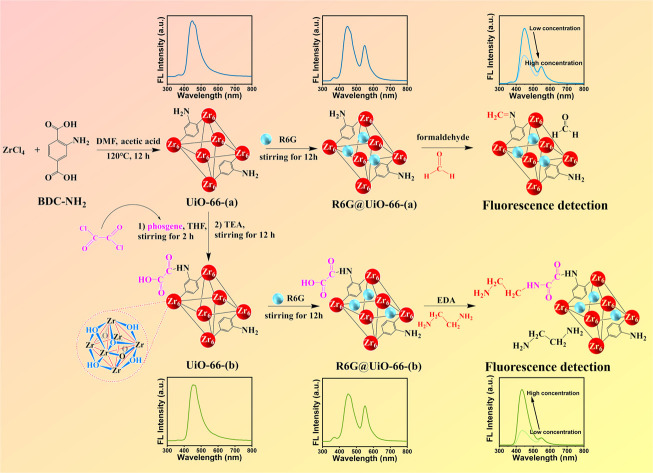

## 1. Introduction

The safety of food and medication has gained prominence, and health issues are gaining more and more attention as people's living standards rise. Small amounts of harmful substances may be produced or introduced during the production, packaging, transportation and storage of food and pharmaceuticals,^1–3^ which may impair product quality and, moreover, cause varying degrees of health damage to consumers.

In addition to being a typical environmental pollutant, formaldehyde is also a useful chemical material that is widely used in the fields of resin manufacturing,^4^ synthetic plastics production,^5^ leather processing^6^ and tissue preservation.^7^ Additionally, in the traditional beer brewing process, sediment can be filtered by adding formaldehyde, which significantly removes polyphenols from wort, reduces wort color, promotes protein flocculation and sediment filtration, and significantly improves the abiotic stability of beer.^8^ Formaldehyde, however, can irritate the intestinal mucosa and result in cancer, perivascular edema, liver and kidney congestion, and pulmonary edema.^9^ Amines, which include organic amines and ammonia, are among the most significant chemical raw materials and are regarded as essential to the growth of the polymer, agrochemical, and pharmaceutical sectors.^10^ For instance, ethylenediamine, a crucial intermediate and precursor in the synthesis of numerous significant chemicals in industrial organic synthesis, different polymers, synthetic insecticides, and so on.^11^ However, amines are highly alkaline, corrosive, and toxic; if not handled properly, they can cause acute kidney injury and tumorigenesis after dermal contact or ingestion into the human body.^12^ Therefore, sensitive and selective detection of formaldehyde and amines is necessary for food and drug determination.

Luminescent methods have gained popularity recently and are being actively pursued in addition to traditional electrochemical, mass spectrometry, and chromatographic methods, which typically have good detection limits but are limited by costly and complex instrumentation, poor portability, and time-consuming analytical procedures.^13,14^ This is because these optical technologies can provide the benefits of dual compatibility between solid and solution media, simplicity, low cost, and quick response time.^15–19^ In this sense, fluorophor@MOF-based sensing materials for luminescence detection of hazardous compounds have drawn more interest recently.

The popularity of porous materials is on the rise because of their attractive properties and diverse applications. Metal–organic frameworks (MOFs) are a new type of porous material that has gained great attention for its simple synthesis, high porosity, and ease of functionalization,^20–22^ making it suitable for potential applications in sensing, gas/liquid phase adsorption, and purification.^23–25^ In particular, a typical zirconium-benzenedicarboxylate MOF, UiO-66, has advantages such as high stability, diverse synthesis methods, easy modification, and wide applicability.^26^ Researchers have made various attempts to improve the selectivity and adsorption efficiency of MOFs, including functionalization. The addition of various functional groups to MOFs has generated a lot of attention since this functionalization can produce desirable qualities and improve MOFs' ability to sense or adsorb targets by chemically attaching to particular kinds of molecules.^27–29^ One of the best post-synthetic covalent modification techniques is direct grafting of functional groups onto the organic linkers of MOFs, and it has been used extensively.^30–32^

UiO-66 can be modified through different post-synthetic methods such as ligand exchange, covalent functionalization, surface modification, and post-synthetic metallation.^33–35^ Among functionalized UiO-66s, amino-functionalized UiO-66 (UiO-66-NH_2_) can be effectively functionalized, and various functional groups can be introduced and modified on the MOF. It has been claimed that reagents including acetic anhydride,^36^ peptide coupling agents,^37^ and aldehydes^38^ can be used to covalently modify the amino groups on MOFs. For instance, UiO-66-NH_2_ can be effectively changed to produce UiO-66-NH_2_ derivatives with various functional groups by combining it with various acid anhydrides,^39,40^ amino acids,^41^ and 1,4-butanedisulfonic acid lactone.^42^ These MOFs have been widely used in gas separation, adsorption, catalysis, and other research fields.^43–47^

**Scheme 1:**
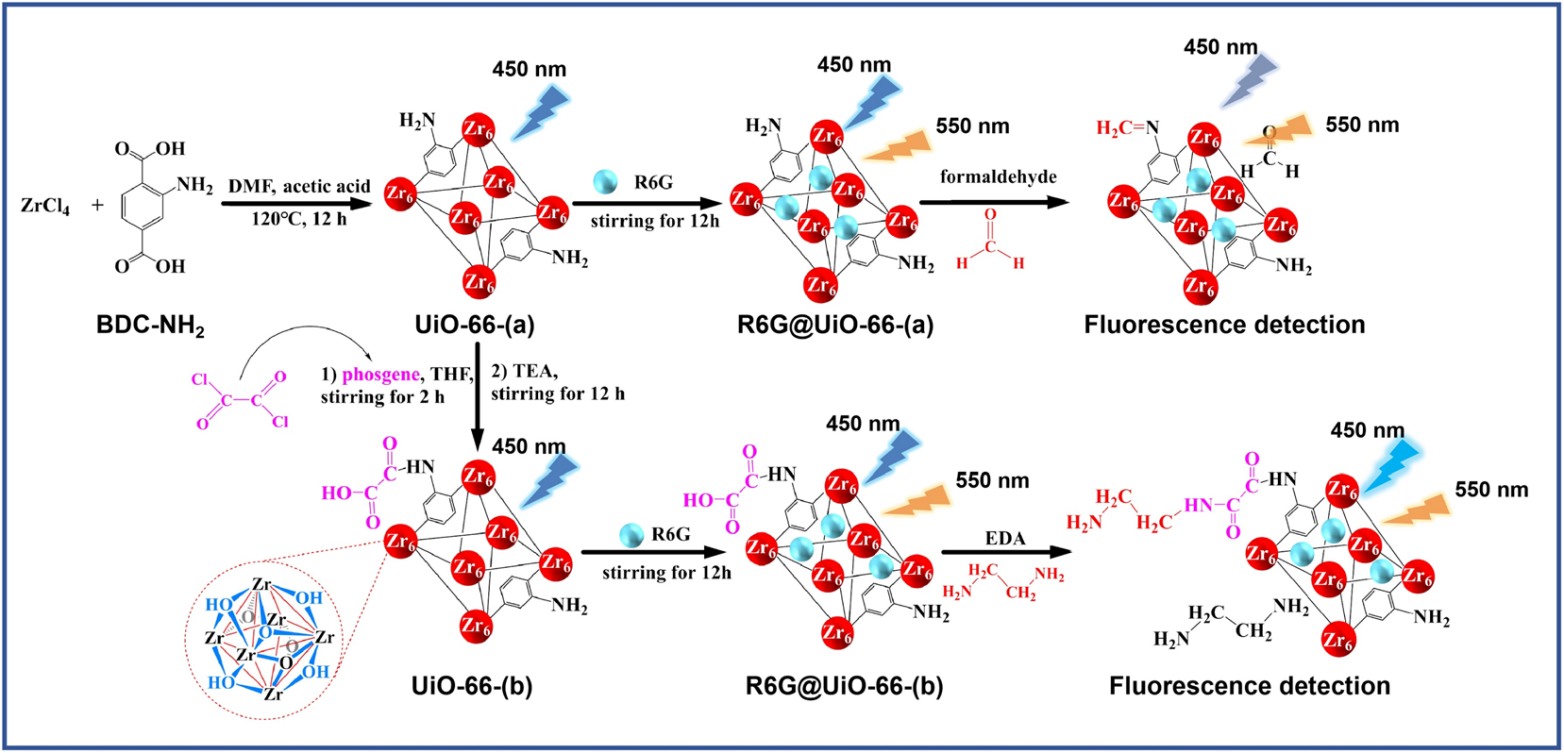
The schematic diagrams of the preparation route of R6G@UiO-66-(a) and R6G@UiO-66-(b) and their application for aldehydes and amines compounds detection, respectively.

Here, UiO-66-NH_2_ (referred to as UiO-66-(a)) was functionalized with phosgene at room temperature to produce functionalized UiO-66 containing –CO and –COOH groups (referred to as UiO-66-(b)). Next, UiO-66-(a) and UiO-66-(b) were doped individually with the fluorescent dye Rhodamine 6G (R6G), yielding R6G@UiO-66-(a) and R6G@UiO-66-(b). These two composites can be utilized to construct ratiometric fluorescent sensors that can detect formaldehyde and amines since they each have two fluorescent emission centers (Scheme 1). Hence, aldehyde-containing formaldehyde can be preferentially bound by UiO-66-(a) based on the condensation process between –CHO and –NH_2_. As well as, UiO-66-(b) has the ability to bind amine compounds selectively, based on the amidation reaction between –COOH and amino groups. R6G@UiO-66-(a) and R6G@UiO-66-(b) alter the initial fluorescence following reactions with formaldehyde and amine chemicals, enabling the quantitative detection of formaldehyde in beer and ethylenediamine in anti-inflammatory agents.

## 2. Experimental

### 2.1 Preparation of UiO-66 and R6G@UiO-66

34.9 mg of ZrCl_4_ and 24.9 mg of 1,4-benzenedicarboxylic acid (BDC) were placed into a 20 mL glass vial, then 10 mL of *N*,*N*-dimethylformamide (DMF) solution containing 2.1 M acetic acid was added. Dispersed the mixture using ultrasound for 1 minute. Closed the glass vial and placed it in an oven at 120 °C for 12 hours to obtain a milky white dispersion. After centrifugation, the collected precipitate was placed in a 60 °C oven to dry and UiO-66 powder was obtained. Mixed 100.0 mg UiO-66 powder with 10.0 mg R6G, the obtained mixture was dispersed in 20 mL pure water, and stirred magnetically at room temperature for 12 hours. After multiple washes with water and centrifugation, the product was collected and dried to obtain R6G@UiO-66 powder.

### 2.2 Preparation of UiO-66-(a) and R6G@UiO-66-(a)

A 20 mL glass vial was filled with 34.9 mg of ZrCl_4_ and 27.2 mg of 2-aminoterephthalic acid (BDC-NH_2_). 10 mL of DMF solution containing 2.1 M acetic acid was then added. The mixture was sonicated for a minute. After sealing the glass vial, it was placed at 120 °C for 12 hours to get a pale yellow solution. Following centrifugation, the precipitate was dried in an oven set at 60 °C to produce UiO-66-(a) powder. Next, 10.0 mg R6G and 100.0 mg UiO-66-(a) powder were combined, the resulting mixture was diluted in 20 mL of water, and it was magnetically agitated for 12 hours at room temperature. To obtain R6G@UiO-66-(a) powder, the product was centrifuged, dried, and repeatedly washed with pure water.

### 2.3 Preparation of UiO-66-(b) and R6G@UiO-66-(b)

The functionalization method was illustrated in Fig. S1,† which has been reported previously.^48^ 300.0 mg UiO-66-(a) powder was slowly added into 16 mL of tetrahydrofuran (THF) containing 320.0 mg of phosgene while stirring thoroughly. After 2 hours, 250.0 mg triethylamine (TEA) was dropwise added to the mixture and continue stirring at room temperature for 12 hours. After centrifugation, collected the yellow-brown precipitate and washed it three times with THF, followed by washing with an adequate amount of pure water. Finally, dried the product in a 60 °C oven to obtain UiO-66-(b) powder. Then 100.0 mg of UiO-66-(b) powder was mixed with 10.0 mg of R6G, then the mixture was dispersed in 20 mL of water, and stirred magnetically at room temperature for 12 hours. After multiple washes with pure water and centrifugation, the product was dried to obtain R6G@UiO-66-(b) powder. (Reminder: phosgene is highly toxic. Please take precautions when using it to prevent poisoning.)

### 2.4 Fluorescence test

2.0 mg of R6G@UiO-66, R6G@UiO-66-(a), and R6G@UiO-66-(b) were taken and dispersed in 10 mL of ethanol or water respectively to obtain corresponding functionalized UiO-66 dispersion to response formaldehyde or amines. During the test, different amounts of formaldehyde or amines are added to the fluorescence response solution and different concentrations of formaldehyde or amines detection solution are obtained after ultrasonic dispersion. Placed the detection solution in a cuvette and recorded the fluorescence signal response using a fluorescence spectrophotometer, with an excitation wavelength of 280 nm.

### 2.5 Preparation of actual samples

For formaldehyde detection, a certain amount of beer sample was taken into a 10 mL centrifuge tube without dilution. 2.0 mg of R6G@UiO-66-(a) material was added and sonicated for 5 min to obtain a detection suspension. For ethylenediamine detection, a certain amount of Caspofungin acetate injection sample was taken into a 10 mL centrifuge tube without dilution. 2.0 mg of R6G@UiO-66-(b) material was added and sonicated for 5 min to obtain a detection suspension. The fluorescence spectra and fluorescence intensity were recorded after shaking.

## 3. Results and discussions

### 3.1 Characterization of functionalized UiO-66s

**Fig. 1:**
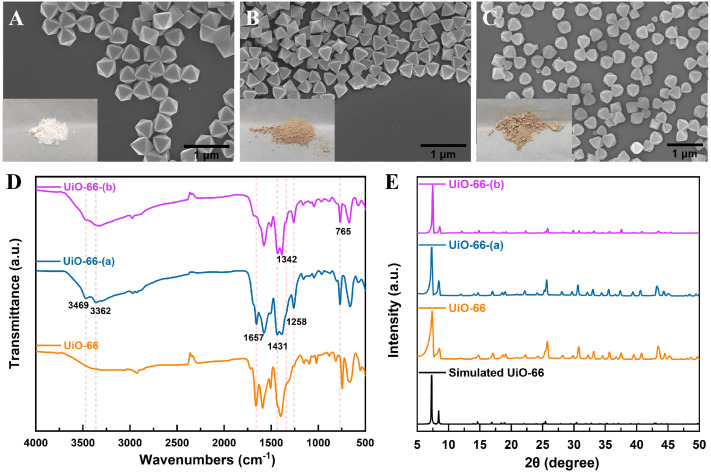
SEM images of (A)UiO-66, (B)UiO-66-(a), (C)UiO-66-(b), the bottom left corner of the SEM image is a photo of the corresponding material sample. (D) FT-IR spectra and (E) XRD patterns of UiO-66, UiO-66-(a) and UiO-66-(b).

Scanning electron microscopy (SEM) was used to characterize the prepared UiO-66, UiO-66-(a), and UiO-66-(b) and corresponding R6G@UiO-66s materials. The results are shown in Fig. 1. It can be seen that UiO-66 (Fig. 1A) exhibits a typical octahedral morphology with uniform particle size of ∼400 nm. The functionalized UiO-66-(a) (Fig. 1B) and UiO-66-(b) (Fig. 1C) show similar octahedral morphology and size as UiO-66. However, UiO-66-(b), due to longer stirring time during functionalization, has some smaller particles. The SEM images of R6G@UiO-66s (Fig. S2A–S2C†) reveal that the surface of UiO-66s materials is distinctly coated with a shell-like layer exhibiting noticeable granularity, indicating that R6G dye is attached to the external surface of UiO-66s materials. The interaction between R6G and UiO-66s materials was investigated by measuring the Zeta potentials of different materials. As displayed in Fig. S3,† R6G carries a positive charge, while the UiO-66s materials exhibit varying degrees of negative charge. Therefore, the interaction between R6G and UiO-66s materials may involve electrostatic interactions. Fig. 1D shows the Fourier-transform infrared spectroscopy (FT-IR) spectra of the three functionalized UiO-66 particles. Absorption peaks at 1258 and 1342 cm^−1^ in the FT-IR spectra of UiO-66-(b) and UiO-66-(a) correspond to C–N stretching,^49^ whereas an absorption band at 1431 cm^−1^ indicates the presence of C–O bonds (carboxylic acid –COOH group) in BDC or BDC-NH_2_.^50^ The successful introduction of –COOH and C=O groups in UiO-66-(b) is confirmed by the presence of a wide band spanning from approximately 1740 to 1700 cm^−1^.^51,52^ The peak at 1657 cm^−1^ may correspond to d to the –CO– of residual DMF molecules trapped inside the pores of UiO-66.^53^ After functionalization, the peak intensity here gradually decreases due to the introduction of –NH_2_ and other functional groups.^54^ After modification, the N–H peak of the amino group around 3500–3300 cm^−1^ show significant changes. For UiO-66-(a), two sharp peaks appear at 3469 and 3362 cm^−1^, corresponding to the doublet of the primary amine. For UiO-66-(b), one sharp peak is observed, which corresponds to the singlet of the secondary amine.^55^ Furthermore, with UiO-66-(b), the addition of additional functional groups to the original –NH_2_ group makes the C–N stretching band less noticeable. Moreover, UiO-66-(a) and UiO-66-(b) spectra show prominent N–H bending absorption peaks at about 765 cm^−1^,^56^ suggesting that functionalized UiO-66 materials were successfully prepared. The ultraviolet-visible (UV-vis) absorption spectra of different UiO-66s were displayed in Fig. S4,† it can be seen that the functionalized UiO-66 materials possess functional groups different from the primitive MOF. According to the Nuclear Magnetic Resonance (^1^H NMR) result in Fig. S5,† the ligand substitution rate in UiO-66-(b) is approximately 27.4% compared to UiO-66-(a). The X-ray diffraction (XRD) spectra of UiO-66, UiO-66-(a), and UiO-66-(b) are displayed in Fig. 1E. The same crystal structures of UiO-66 and the two functionalized UiO-66 materials suggest that there were no notable changes to UiO-66's crystal structure following functionalization.

**Fig. 2:**
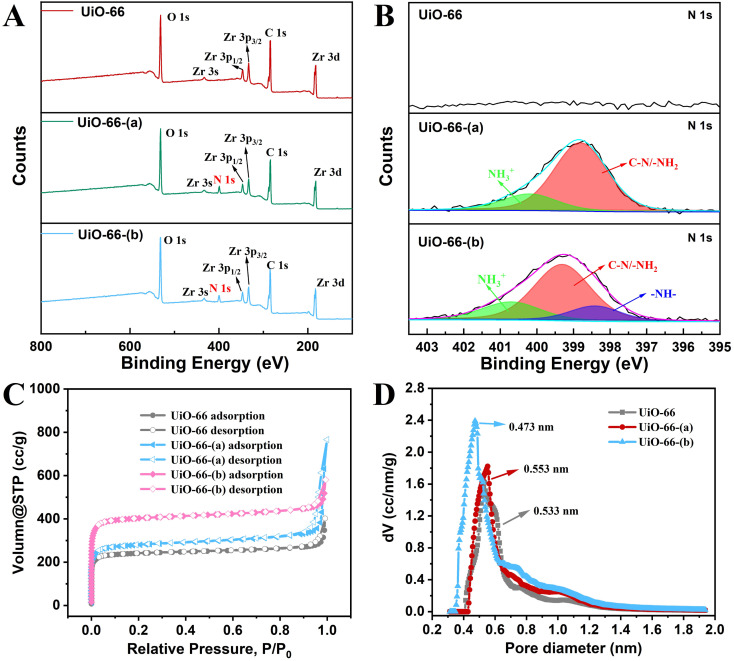
(A)The XPS survey spectra and (B) N 1s XPS spectra of the three UiO-66s materials. (C) N_2_ adsorption isotherms and (D) pore size distribution of UiO-66, UiO-66-(a) and UiO-66-(b).

The XPS survey spectra and N 1s XPS spectra of the three UiO-66s materials were exhibited in Fig. 2A and B. From the N 1s XPS spectrum, it can be seen that there is no N element in UiO-66, while UiO-66- (a) exhibits C–N/-NH_2_ groups peaks (398.8 eV) attributed to the ligands, and another peak at 400.2 eV corresponding to protonated –NH_3_^+^ species. In UiO-66- (b), a newly formed peak (–NH– moieties) can be observed at 398.4 eV, indicating that partial primary amines have reacted with phosgene. Thus, it was confirmed that the functional groups of carboxylic acid were chemically bonded with the amine group. The isothermal N_2_ adsorption/desorption curves of the three UiO-66s materials are displayed in Fig. 2C. Each of the three exhibits type I isotherms, a sign of microporous structures.^57^ The estimated specific surface areas of Brunauer–Emmett–Teller (BET) are 1624.9 m^2^ g^−1^ (UiO-66-(b)), 1123.2 m^2^ g^−1^ (UiO-66-(a)), and 970.3 m^2^ g^−1^ (UiO-66). The reduction in particle size during the functionalization process may be the cause of UiO-66-(b)'s greater specific surface area when compared to UiO-66-(a).^58^ The distribution of pore sizes for the three UiO-66 materials is depicted in Fig. 2D. The results reveal that UiO-66 and UiO-66-(a) have similar pore sizes, whereas UiO-66-(b) has the smallest pore size, mostly because of the extra functional groups attached inside its pores. The pore size distribution profiles of the R6G@UiO-66s materials was also supplied in Fig. S6.† The results show that the original pore sizes of UiO-66s decreased after combining with R6G dye, demonstrating that R6G dye molecules may also have entered the pores of UiO-66s. Thermogravimetric analysis (TGA) was used to examine the thermal stabilities of the UiO-66s materials. The findings displayed in Fig. S7† suggest that the addition of functional groups marginally decreased the thermal stabilities of the UiO-66s materials. In general, functionalizations reduced the thermal stability of UiO-66s materials.^48^

**Fig. 3:**
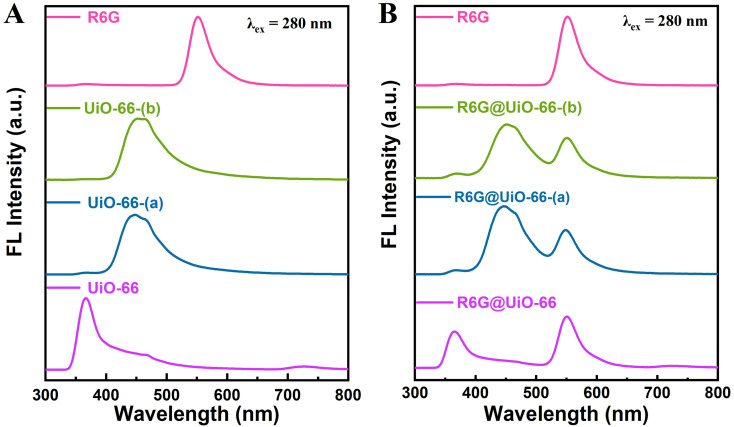
(A) Fluorescence emission spectra of UiO-66, UiO-66-(a), UiO-66-(b) and R6G, (B) fluorescence emission spectra of R6G@UiO-66, R6G@UiO-66-(a) and R6G@UiO-66-(b) after doping with R6G, *λ*_ex_ = 280 nm.

UiO-66, UiO-66-(a), and UiO-66-(b) themselves can emit fluorescence when excited at a wavelength of 280 nm, as shown in Fig. 3A. After doping with R6G fluorescent molecules, R6G@UiO-66, R6G@UiO-66-(a), and R6G@UiO-66-(b) were dispersed in ethanol. At the excitation wavelength of 280 nm, all three functionalized particles produced dual emission peak fluorescence containing the R6G emission peak (∼550 nm) (Fig. 3B). By using the R6G fluorescence emission peak as a reference, a ratiometric fluorescence sensor was constructed. UiO-66, UiO-66-(a), and UiO-66-(b) undergo structural changes when in contact with formaldehyde or ammonia molecules, and accordingly, the fluorescence intensity changes. The relative change in fluorescence intensity before and after the reaction was used to quantitatively detect various volatile compounds. Furthermore, PXRD was employed to characterize the phase purity of the three UiO-66s and R6G@UiO-66s materials (Fig. S8†). The peaks in the MOFs' XRD patterns closely resemble the UiO-66 simulated pattern. According to the XRD patterns, all the UiO-66s and R6G@UiO-66s materials are isostructural with the parent UiO-66, demonstrating that following doping with Rhodamine 6G dye, MOFs' crystallinity and structure were preserved.

### 3.2 Fluorescence stability of functionalized R6G@UiO-66s

**Fig. 4:**
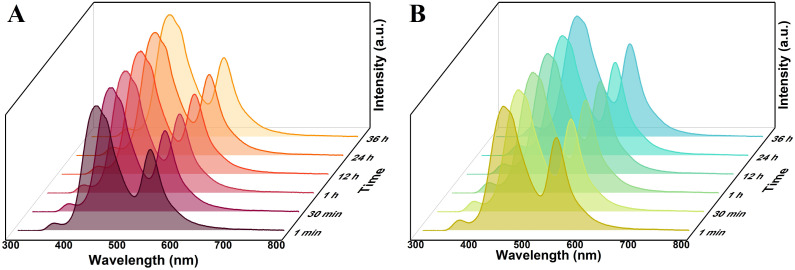
The fluorescence stability of (A) R6G@UiO-66-(a) and (B) R6G@UiO-66-(b) in ethanol solvent, *λ*_ex_ = 280 nm.

Before using functionalized R6G@UiO-66s, the fluorescence stability of the three types of R6G@UiO-66s needs to be determined. R6G@UiO-66, R6G@UiO-66-(a), and R6G@UiO-66-(b) were dispersed in ethanol solvent to examine the changes in fluorescence emission over time. The results, as shown in Fig. S9A,† indicate that within 30 minutes, the emission peak intensity of UiO-66 in R6G@UiO-66 increased with time. This may be due to ethanol further binding with the unsaturated UiO-66 sites, forming a more stable UiO-66 structure and increasing the fluorescence emission peak of UiO-66. This also suggests that the fluorescence emission of UiO-66 in ethanol solvent is unstable and not suitable for detecting other volatile compounds molecules. On the other hand, the fluorescence of R6G@UiO-66-(a) (Fig. 4A) and R6G@UiO-66-(b) (Fig. 4B) in ethanol remained stable within 36 hours, indicating that the functionalization of UiO-66 increased its stability. These two functionalized R6G@UiO-66s can be used to detect other reactive volatile organic compounds (VOCs) molecules.

**Fig. 5:**
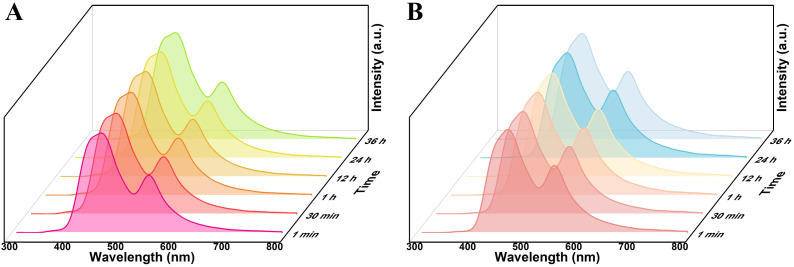
The fluorescence stability of (A) R6G@UiO-66-(a) and (B)R6G@UiO-66-(b) in aqueous solution, *λ*_ex_ = 280 nm.

The fluorescence emission spectra of the three types of particles, R6G@UiO-66, R6G@UiO-66-(a), and R6G@UiO-66-(b), dispersed in the commonly used solvent water were also investigated. It can be seen that within 30 minutes, the fluorescence emission peak intensity of UiO-66 in R6G@UiO-66 decreases with increasing time (Fig. S9B†), indicating that water molecules have a quenching effect on the fluorescence of UiO-66, which is consistent with previous reports.^59^ This suggests that the fluorescence emitted by R6G@UiO-66 in water is also unstable and not suitable for detecting other volatile compounds molecules. However, the fluorescence of R6G@UiO-66-(a) (Fig. 5A) and R6G@UiO-66-(b) (Fig. 5B) in water remains stable within 36 hours, indicating that these two functionalized R6G@UiO-66 aqueous solutions can be used to detect other reactive volatile compounds molecules. We further compared the fluorescence quantum yields and lifetimes of UiO-66, UiO-66-(a) and UiO-66-(b) (Fig. S10†). The results demonstrated that functionalization increased the quantum yield from 3.8% to 4.5% and 10.5%, and the fluorescence lifetime were extended from 0.42 ns to 0.54 ns and 4.50 ns, respectively, which collectively enhanced UiO-66's fluorescence performance. In addition, although UiO-66 is unstable in both ethanol and water, the fluorescence emitted by R6G@UiO-66 under excitation wavelength of 280 nm shows different trends. This suggests that R6G@UiO-66 may be used to determine ethanol and water, which will be further studied in future work.

### 3.3 Fluorescence method for the detection of volatile substances

**Fig. 6:**
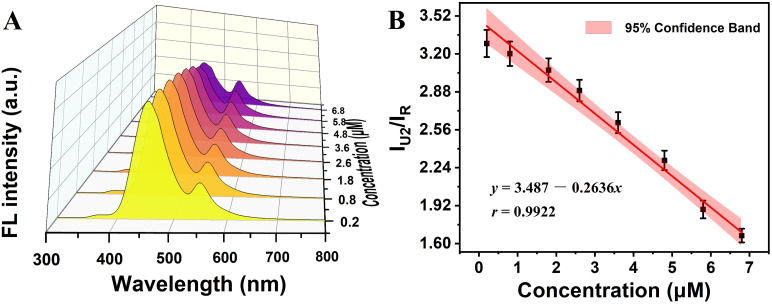
(A) The fluorescence response of R6G@UiO-66-(a) to different concentrations of formaldehyde in ethanol. (B) The concentration of formaldehyde has a linear relationship with the relative fluorescence intensity of R6G@UiO-66-(a), *λ*_ex_ = 280 nm.

Formaldehyde was detected using R6G@UiO-66-(a) in ethanol solvent. The simple aldehyde-amine condensation reaction of the –NH_2_ group in UiO-66-(a) with the –CHO group of the formaldehyde caused a change in the fluorescence intensity emitted by the original UiO-66-(a).^60^ The outcomes are displayed in Fig. 6A. As can be observed, R6G@UiO-66-(a) reacted with formaldehyde to boost UiO-66-(a)'s fluorescence. A negative correlation linear relationship between the fluorescence intensity and the formaldehyde concentration was obtained over a specific concentration range, as illustrated in Fig. 6B. The formaldehyde detection ranges were 0.2–6.8 μM with a detection limit (LOD) of 0.058 μM. This was achieved by using the concentration of formaldehyde as the horizontal coordinate and UiO-66-(a) fluorescence intensity/R6G fluorescence intensity (*I*_U2_/*I*_R_) as the vertical coordinate.

**Fig. 7:**
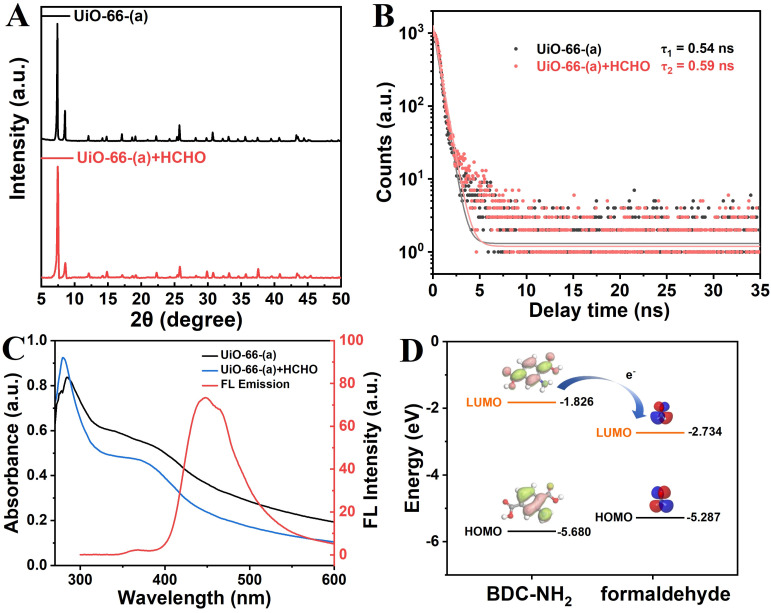
(A) XRD patterns. (B) Time-resolved decay curves of UiO-66-(a) in the absence and presence of formaldehyde. (C) Overlap of emission spectra of UiO-66-(a) and absorbance spectra of free formaldehyde. (D) HOMO–LUMO energy level of ligand BDC-NH_2_ and formaldehyde.

The following explains how formaldehyde quenches the fluorescence of UiO-66-(a). The XRD pattern of UiO-66-(a) was unaltered both before and after formaldehyde quenching, as seen in Fig. 7A. This suggested that the integrity of the crystal structure was unaffected by the formaldehyde addition. Thus, structure collapse could not be the reason of the fluorescence's quenching.^61^ A new absorption peak at about 380 nm was produced by the reaction with formaldehyde, as illustrated in Fig. 7C. This mean that after formaldehyde attached to UiO-66-(a), the formation of C=N bond between carbonyl group and amino-group promotes the electron transition from the amine-containing chromophore to Zr-oxo cluster.^62^ This new absorption spectra partially overlapped with the fluorescence emission spectrum of UiO-66-(a). Fluorescence resonance energy transfer (FRET) from the ligand fluorophore to the non-emitting fluorophore may therefore be the partial cause of the fluorescence quenching.^63^ The fluorescence lifetime of UiO-66-(a) did not change significantly before and after formaldehyde was added, as seen in Fig. 7B. This suggests that the quenching process described above is primarily caused by static quenching, which results from the combination of the quencher and the fluorophore to form non-luminescent ground state complexes. Furthermore, Fig. 7D shows that the lowest unoccupied molecular orbital (LUMO) of BDC-NH_2_ is higher than the energy level of formaldehyde, which accelerate the transfer of electron from the LUMO of the ligand to the LUMO of formaldehyde, resulting in fluorescence quenching. This demonstrates the existence of photoinduced electron transfer (PET) between UiO-66-(a) and formaldehyde.^64^ As demonstrated above, the fluorescence quenching of UiO-66-(a) by formaldehyde involves multiple quenching mechanisms and is a complex process.

Meanwhile, R6G@UiO-66-(b) was used for the detection of three different amine (ammonia, methylamine and ethylenediamine) organic volatiles in aqueous system. The fluorescence intensity emitted by the original UiO-66-(b) was changed due to the easy condensation reaction between the –C=O group and –COOH group in R6G@UiO-66-(b) with the –NH_2_ group in the amine molecules,^65^ and the results are shown in Fig. S11A, S12A† and 8A. It can be seen that the fluorescence of UiO-66-(b) was increased after reaction with all three amine organic volatiles, but the increase degree of fluorescence was different. Taking the concentration of the amine organic volatiles as the horizontal coordinate, the fluorescence intensity of UiO-66-(b)/the fluorescence intensity of R6G (*I*_U3_/*I*_R_) as the vertical coordinate, the linear relationship between the concentration of amine organic compounds and the fluorescence intensity in a certain concentration range was obtained, and the detection concentration ranges were 0.05–1.2 μM, 0.01–0.8 μM and 0.005–0.275 μM for ammonia (LOD of 0.022 μM), methylamine (LOD of 0.0043 μM) and ethylenediamine (LOD of 0.0017 μM), respectively, as shown in Fig. S11B, S12B† and 8B. It can be seen that the fluorescent sensor is more and more sensitive to VOCs with the increase of –NH_2_ groups in amine organic compounds, *i.e.*, the order of detection sensitivity (S) is: S (ethylenediamine) > S (methylamine) > S (ammonia). This may be due to the fact that ethylenediamine has more –NH_2_ groups involved in the condensation reaction for the same amount of VOCs molecules.

**Fig. 8:**
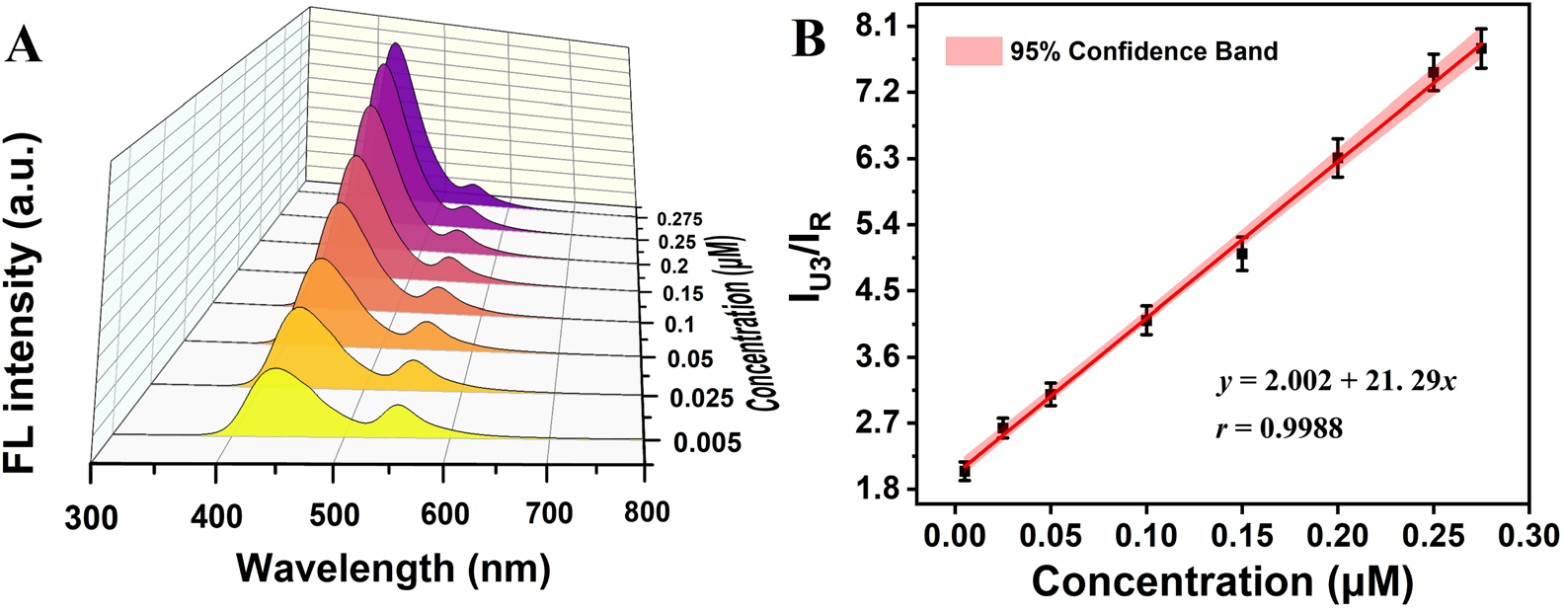
(A) The fluorescence response of R6G@UiO-66-(b) to different concentrations of ethylenediamine in aqueous solution. (B) The concentration of ethylenediamine has a linear relationship with the relative fluorescence intensity of R6G@UiO-66-(b), *λ*_ex_ = 280 nm.

**Fig. 9:**
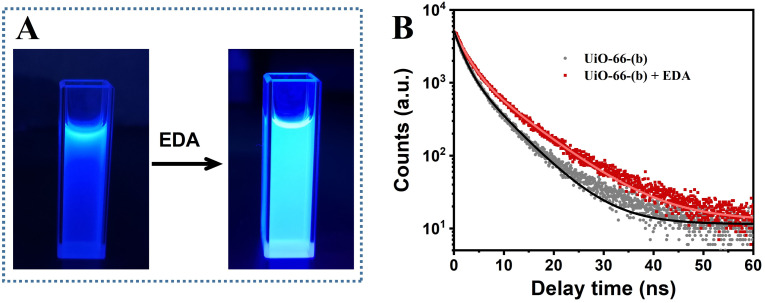
(A) The fluorescence of UiO-66-(b) dispersion before and after the addition of ethylenediamine (EDA) under the irradiation of ultraviolet analyzer. (B) Time-resolved fluorescence decay curves of UiO-66-(b) before and after the addition of ethylenediamine.

It has been reported that some amine organic molecules can be used as surface passivators in combination with carbon quantum dots (CDs) modified with a large number of carboxyl groups on the surface can significantly enhance the emission of CDs.^66,67^ In this work, amine organic molecules (*e.g.*, ethylenediamine) were added to UiO-66-(b) dispersions with surface-modified carboxyl groups, and the –NH_2_ groups of the amine organic molecules were combined with –COOH on the surface of the functionalized MOFs in a condensation reaction, which led to a significant enhancement of fluorescence of UiO-66-(b), as shown in Fig. 9A. The FTIR spectra and UV-vis spectra of UiO-66-(b) before and after contact with ethylenediamine (EDA) were displayed in Fig. S13 and S14,† they all confirmed the reaction between amine groups of EDA and carboxyl groups in UiO-66-(b). The fluorescence lifetimes of UiO-66-(b) before and after the addition of ethylenediamine were measured to investigate the effect of ethylenediamine on the fluorescence properties. As shown in Fig. 9B, the average fluorescence lifetime of UiO-66-(b) was 4.50 ns before the addition of ethylenediamine, while the fluorescence decay time increased to 6.12 ns after the addition of ethylenediamine, and the increase in the fluorescence lifetime indicated that the ethylenediamine molecules bound to the surface of the MOFs could act as electron donors to enhance the fluorescence intensity of UiO-66-(b).^68^

### 3.4 Selectivity of the fluorescence detection method

**Fig. 10:**
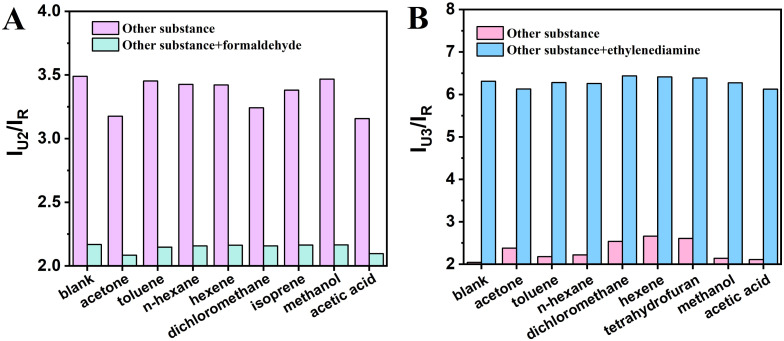
Fluorescence response of (A) R6G@UiO-66-(a) and (B) R6G@UiO-66-(b) to several common volatile organic compounds and their coexistence with analytes.

Seven common VOCs molecules were detected using these two constructed ratiometric sensors, respectively. For the R6G@UiO-66-(a) fluorescent sensor, acetone (20 μM), toluene (20 μM), *n*-hexane (20 μM), hexene (20 μM), dichloromethane (20 μM), isoprene (20 μM), methaonl (20 μM), acetic acid (20 μM) mixed with formaldehyde (5 μM) were detected; for the R6G@UiO-66-(b) fluorescent sensor, acetone (20 μM), toluene (20 μM), hexane (20 μM), dichloromethane (20 μM), hexene (20 μM), tetrahydrofuran (20 μM), methaonl (20 μM), acetic acid (20 μM), mixed with ethylenediamine (0.2 μM) were also measured. The test results are shown in Fig. 10, R6G@UiO66-(a) exhibits virtually unchanged fluorescence intensity in the presence of other high-concentration VOCs; however, upon the addition of a small amount of formaldehyde, the fluorescence intensity significantly decreases. This indicates that the R6G@UiO66-(a) fluorescent probe demonstrates excellent selectivity toward formaldehyde (Fig. 10A). For the UiO-66-(b) fluorescent probe, even high concentrations of other VOCs do not induce changes in its fluorescence intensity. However, when a small amount of ethylenediamine is introduced, the fluorescence intensity increases significantly. This confirms the UiO-66-(b) fluorescent probe's great selectivity for ethylenediamine (Fig. 10B), and their selectivity is attributed to the functional groups modified on the UiO-66 material.

### 3.5 Actual samples detection

The detection application of these fluorescence probes based on R6G@UiO-66-(a) and R6G@UiO-66-(b) for formaldehyde in beer samples and ethylenediamine in caspofungin acetate injection sample were investigated.

A kind of beer was provided by a local beer manufacturer. The beer samples without dilution were spiked with standard formaldehyde solutions at three different levels of 2, 4 and 6 μM for fluorescence analysis. The spiking and recovery results were displayed in Table 1. The result exhibits the formaldehyde recoveries utilizing R6G@UiO-66-(a) probe, which ranged from 97.3% to 108.5%. The reproducibility of each spiked sample was assessed thrice. As a result, the aforementioned findings demonstrated that the R6G@UiO-66-(a) probe could be used to evaluate actual liquor samples and accurately detect the formaldehyde content.

**Table 1:** Formaldehyde detection and spiked recovery results in beer samples by R6G@UiO-66-(a) fluorescence probe

Sample	Spiked (μM)	Found (μM)	Recovery (%)	RSD (%) (*n* = 3)
Beer	0.00	—	—	—
	2.00	1.92	96.0	2.37
	4.00	4.07	101.8	3.58
	6.00	6.33	105.5	2.79

Caspofungin acetate is a kind of medicine, can effectively prevent and treat infections caused by filamentous fungi and yeast, as well as pneumonia caused by Pneumocystiscarinii. Ethylenediamine, as one of the raw materials for the synthesis of carbaprofen acetate, may be residual. Caspofungin acetate injection samples were purchased from a pharmacy nearby Yan'an University. The caspofungin acetate samples without dilution were spiked with standard ethylenediamine solutions at three different levels of 0.08, 0.16 and 0.24 μM for fluorescence analysis. Spiking and recovery studies were also performed, the results were displayed in Table 2. The result shows the range of ethylenediamine recoveries obtained with R6G@UiO-66-(b) probe, which was 96.4% to 103.3%. The reproducibility of each spiked sample was assessed thrice. As a result, the aforementioned findings demonstrated that the R6G@UiO-66-(b) probe could be used to evaluate actual medical samples and accurately detect the ethylenediamine concentration.

**Table 2:** Ethylenediamine detection and spiked recovery results in caspofungin acetate samples by R6G@UiO-66-(b) fluorescence probe

Sample	Spiked (μM)	Found (μM)	Recovery (%)	RSD (%) (*n* = 3)
Caspofungin acetate	0	—	—	—
	0.08	0.08	96.4	2.26
	0.16	0.15	96.9	3.89
	0.24	0.25	103.3	3.17

Finally, comparisons of present functionalized R6G@UiO-66 fluorescent probes with other different methods for formaldehyde and ethylenediamine detection are given in Tables S1 and S2.† We found that both the two functionalized R6G@UiO-66 exhibit a low detection limit. In addition, these two fluorescent probes showed excellent fluorescence performance in complex medium and can be easily detected, making them more potential for practical testing.

## 4. Conclusion

In this work, two MOFs materials, UiO-66-(a) and UiO-66-(b), were prepared by using functionalized organic ligands and post-synthesis modification, respectively, followed by combining with the doping method to encapsulate the fluorescent molecule Rhodamine 6G (R6G) into these two MOFs materials to obtain R6G@UiO-66-(a) and R6G@UiO-66-(b) fluorescent probe, respectively. The ratiometric fluorescent sensors constructed with these two fluorescent probe were utilized to detect formaldehyde and amine substances, respectively. Based on the aldehyde-amine condensation reaction of –NH_2_ in R6G@UiO-66-(a) with aldehyde –CHO and the condensation reaction of –C=O and –COOH in R6G @UiO-66-(b) with amine –NH_2_, the two constructed ratiometric fluorescent sensors showed good selectivity for formaldehyde and ethylenediamine, respectively. It is promising to construct sensor devices from these two materials for the detection of volatile harmful substances in food or medicines.

## Data availability

The data supporting this article have been included as part of the ESI.†

## Conflicts of interest

There are no conflicts of interest to declare.

## Supplementary Material

RA-015-D5RA01251A-s001
