# Supplementary material for: Two ratiometric fluorescent sensors originating from functionalized R6G@UiO-66s for selective determination of formaldehyde and amine compounds
Source: RSC Adv. 2025 May 6;15(18):14532–44. doi: 10.1039/d5ra01251a (PMC12054354; doi:10.1039/d5ra01251a)
Supplement: RA-015-D5RA01251A-s001 [file RA-015-D5RA01251A-s001.pdf]

Supplementary Information (SI) for RSC Advances.
This journal is © The Royal Society of Chemistry 2025

Two ratiometric fluorescent sensors originated from functionalized R6G@UiO-66s for selective determination of formaldehyde and amine compounds

Wanqiao Bai*[1], Zhuojun Zhao[1], Ting Zhang, Hongmei Chai, Loujun Gao*

Shaanxi Key Laboratory of Chemical Reaction Engineering, College of Chemistry and Chemical Engineering, Yan'an University, Yan'an 716000, P. R. China

*Corresponding author.
*E-mail addresses: glj@yau.edu.cn (L. Gao),
baiwanqiao@yau.edu.cn (W. Bai).
[1]The authors contributed equally to this work.

1. Reagents and Instruments

Zirconium chloride ($ZrCl_4$), 1,4-phenylenedicarboxylic acid (BDC), 2-aminoterephthalic acid (BDC-NH_2), N, N-dimethylformamide (DMF), Rhodamine 6G (R6G), acetic acid, oxalyl chloride, tetrahydrofuran (THF), ethanol, ammonia, ethylenediamine (EDA), triethylamine (TEA), formaldehyde, glyoxal, glutaraldehyde, methylamine, carbofuran acetate, acetone, toluene, n-hexane, hexene, dichloromethane, isoprene, all reagents used are of analytical grade. Beer and injection samples were purchased from local supermarkets and clinics, respectively.

The S-4800 scanning electron microscope (Hitachi, Japan) was employed to characterize the morphology of functionalized UiO-66s. The crystal structure type of the material was determined using the D/max2500 X-ray diffractometer (Rigaku, Japan). The TENSOR-27 Fourier transform infrared spectrometer (Brooke, Germany) was used to obtain functional group information of the material. The adsorption type, specific surface area, pore size distribution, and other information of the material were obtained by ASAP 2020HD88 BET fully automatic physical adsorption instrument (Mack, USA), and the fluorescence response was measured using RF-5301PC fluorescence spectrophotometer (Shimadzu, Japan). The fluorescence lifetime was measured by FLS1000 steady-state/transient fluorescence spectrometer (Edinburgh, UK). The nuclear magnetic resonance (NMR) spectrum was obtained using a 400 MHz nuclear magnetic spectrometer (Bruker, Germany). TG analysis was performed with an STA 6000 thermogravimetric analyzer (PerkinElmer, USA). The XPS spectrum was obtained using a K-Alpha X-ray photoelectron spectrometer (Thermo Scientific, USA).

2. The functionalization route of UiO-66-(b)

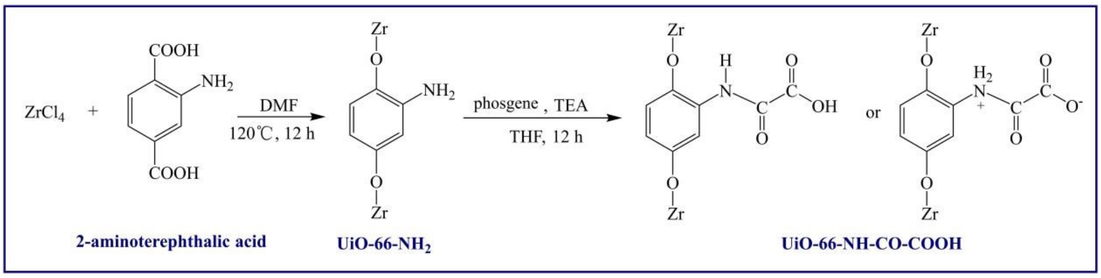

Figure S1 The functionalization route of UiO-66-(b).

3. SEM images of R6G@UiO-66s materials

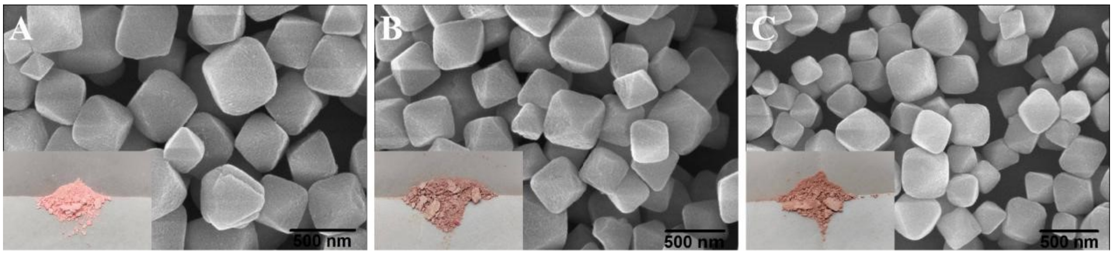

Fig. S2 SEM images of (A)R6G@UiO, (B)R6G@UiO-(a) and (C)R6G@UiO-(b), the bottom left corner of the SEM image is a photo of the corresponding material sample.

4. The Zeta potentials of different materials

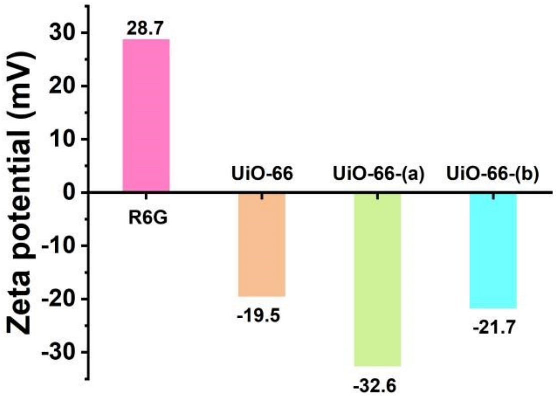

Fig. S3 The Zeta potential of R6G, UiO-66, UiO-66-(a) and UiO-66-(b).

5. The ultraviolet-visible absorption spectra of different UiO-66s

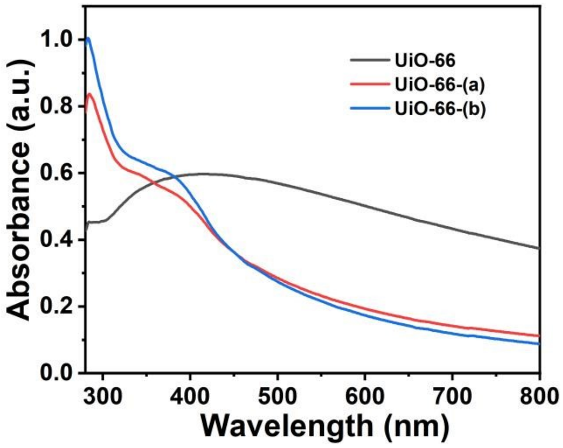

Fig. S4 The ultraviolet-visible (UV-Vis) absorption spectra of different UiO-66s materials.

6. ^{1}H NMR spectra of the three UiO-66s materials

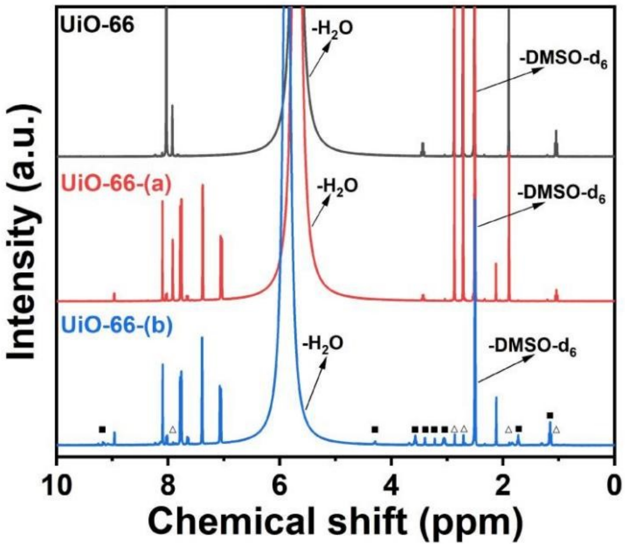

Fig. S5 ^{1}H NMR spectra of UiO-66, UiO-66-(a) and UiO-66-(b).

In order to determine the carboxylic acid groups formed in UiO-66-(b), Nuclear Magnetic Resonance (NMR) tests on the three types of UiO-66s materials were conducted to calculate the yield of modified materials after synthesis. For digestion, 10 mg of UiO-66s material sample was added to a mixture of 30 μL HF and 570 μL DMSO-d_6. The ^{1}H NMR spectrum results are shown in **Fig. S5**, and the proton types and distribution of H atoms varies in different UiO-66s materials (the peaks marked with solid blocks represent newly emerged proton types, and the peaks marked with hollow triangles represent disappeared or weakened proton types). After calculation, the ligand substitution rate in UiO-66-(b) is approximately 27.4% compared to UiO-66-(a), indicating that approximately 27.4% of the amine groups in the UiO-66-(a) structure formed carboxyl groups.

7. Pore size distribution of R6G@UiO-66s materials

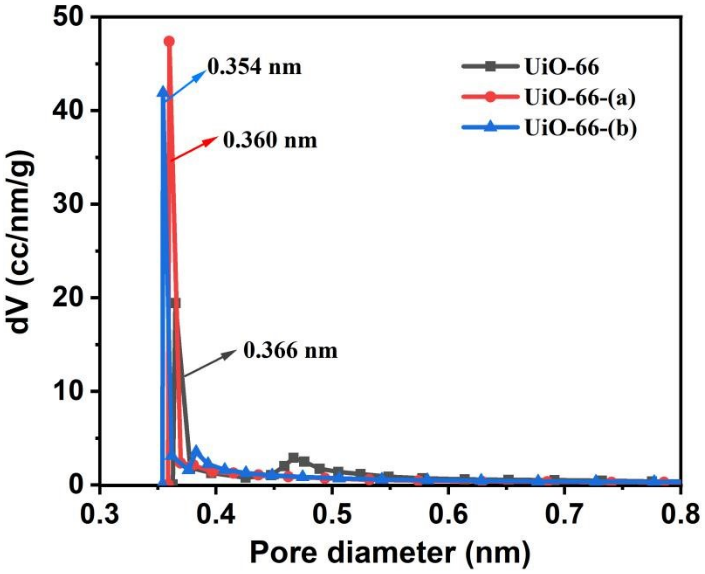

Fig. S6 Pore size distribution of R6G@UiO-66, R6G@UiO-66-(a) and R6G@UiO-66-(b).

8. TGA patterns of the three UiO-66s materials

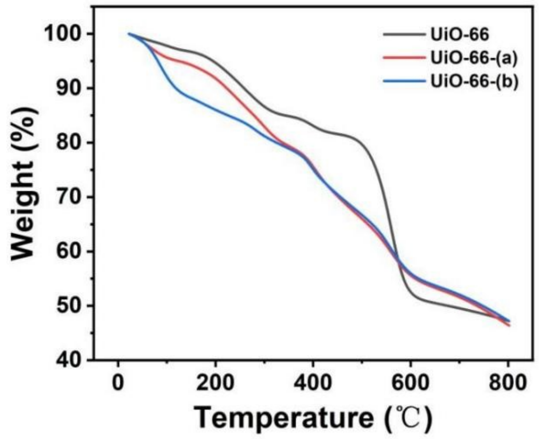

Fig. S7 TGA patterns of the three UiO-66s materials.

The thermal stabilities of the UiO-66s materials were analyzed by thermogravimetric analysis (TGA). The results shown in **Fig. S7** indicate that the thermal stabilities of the UiO-66s materials were slightly reduced with the introduction of functional groups. The thermal stability of UiO-66s materials generally decreased with functionalizations (*Chemical Engineering Journal, 331 (2018) 124-131*).

.

9. PXRD patterns of UiO-66s and R6G@UiO-66s materials.

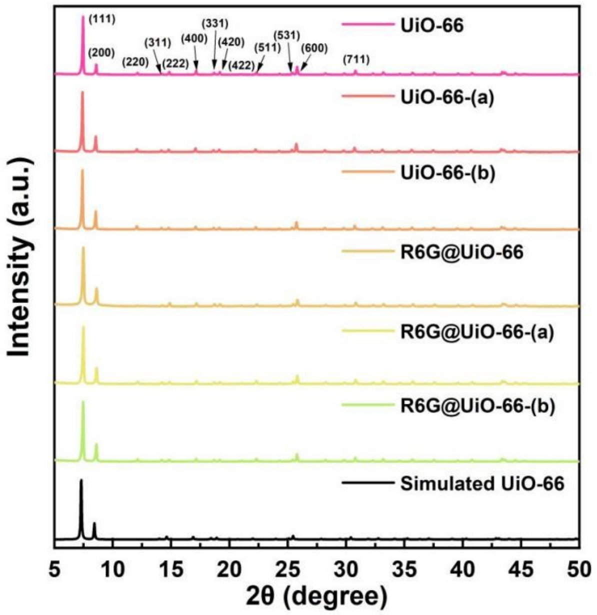

Fig. S8 PXRD patterns of UiO-66s and R6G@UiO-66s materials.

The phase purity of the three UiO-66s and R6G@UiO-66s materials was characterized by PXRD (**Fig. S8**). The peaks in the PXRD patterns of the MOFs match well with the simulated pattern of UiO-66 . The PXRD patterns indicated that the UiO-66-(a) and UiO-66-(b) are isostructural with the parent UiO-66. Also, the patterns showed that the crystallinity and structure of MOFs were retained after doping with Rhodamine 6G dye. (*ACS Applied Materials & Interfaces, 12 (2020), 25221-25232*) All of the XRD patterns clearly showed the reflection peaks of 2θ = 7.36, 8.50, 12.06, 14.12, 14.77, 17.06, 18.59, 19.08, 20.96, 22.20, 25.35, 25.69, and 30.68, which corresponded, respectively, to (111), (200), (220), (311), (222), (400), (331), (420), (422), (511), (531), (600), and (711) Bragg planes as the same those of the simulated and reported UiO-66 (*Journal of Molecular Liquids, 325 (2021) 115228*). This result was proved that these three MOF structures are the same and the pristine UiO-66 remained obviously intact after the postsynthetic modifications.

10. The fluorescence emission spectra of R6G@UiO-66 in different solvents.

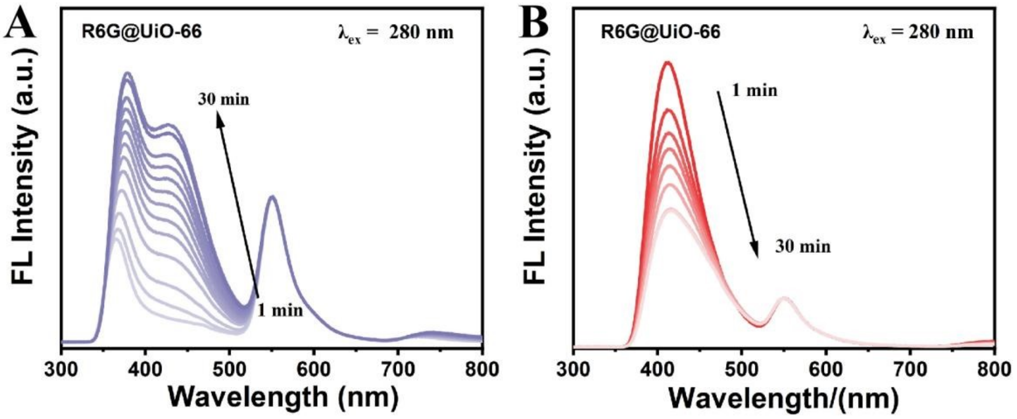

Fig. S9 The fluorescence stability of R6G@UiO-66 in (A) ethanol solvent and (B) aqueous solution.

11. Time-resolved fluorescence decay curves of different UiO-66s materials

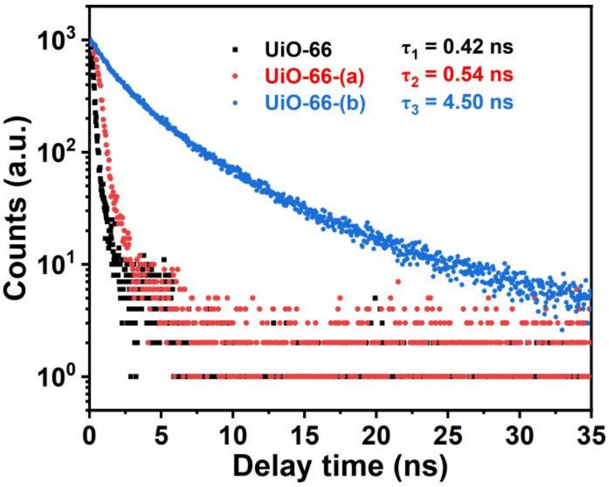

Fig. S10 Time-resolved fluorescence decay curves of different UiO-66s materials.

12. The fluorescence response of R6G@UiO-66-(b) to ammonia

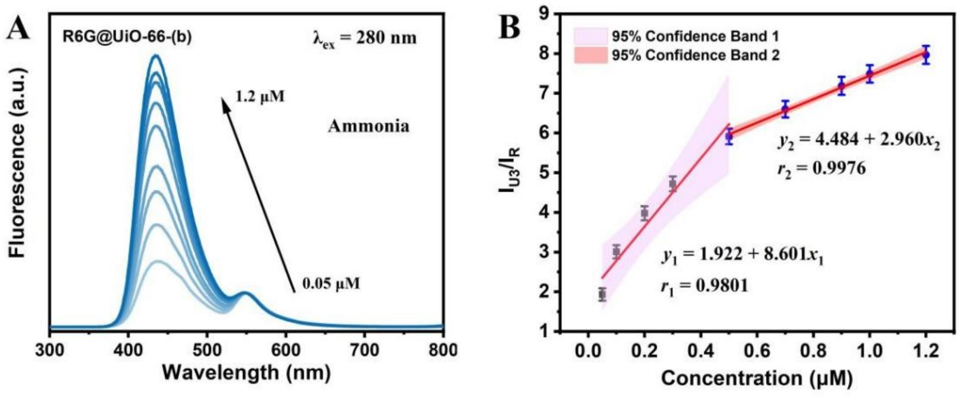

Figure S11 (A) The fluorescence response of R6G@UiO-66-(b) to different concentrations of ammonia in aqueous solution. (B) The concentration of ammonia has a linear relationship with the relative fluorescence intensity of R6G@UiO-66-(b). The excitation wavelength is 280 nm.

13. The fluorescence response of R6G@UiO-66-(b) to methylamine

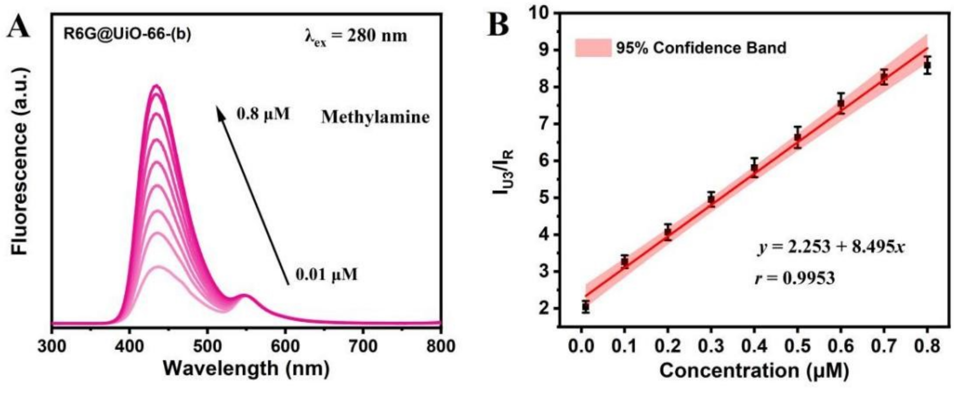

Fig. S12 (A) The fluorescence response of R6G@UiO-66-(b) to different concentrations of methylamine in aqueous solution. (B) The concentration of methylamine has a linear relationship with the relative fluorescence intensity of R6G@UiO-66-(b). The excitation wavelength is 280 nm.

14. FTIR spectra of UiO-66-(b) before and after the reaction with EDA

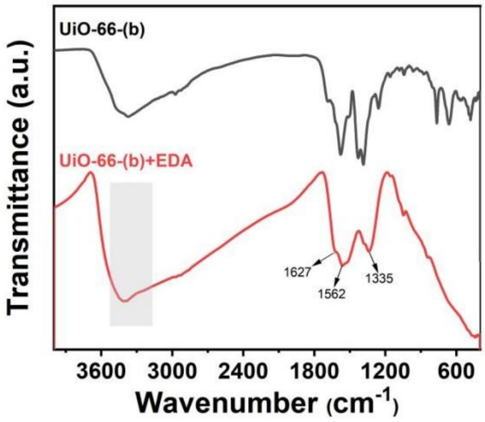

Fig. S13 FTIR spectra of UiO-66-(b) before and after the reaction with ethylenediamine (EDA).

The FTIR spectra of UiO-66-(b) before and after the reaction with ethylenediamine (EDA) were compared in **Fig. S13** . It can be seen that there were significant changes after the reaction with ethylenediamine. The broad peak enhanced at 3100-3500 cm^{-1} is attributed to primary amines, secondary amines, and amides. A new broad peak was observed at 1562 cm^{-1} along with a weak band at 1627cm^{-1}, attributing to the bending of N–H and the stretching of C=O on the secondary amide group. Another new enhanced peak at 1335 cm^{-1} indicates the formation of C-N bonds, confirming the bonding between amine groups of EDA and carboxyl groups in UiO-66-(b).

15. UV-vis spectra of UiO-66-(b) before and after contact with EDA

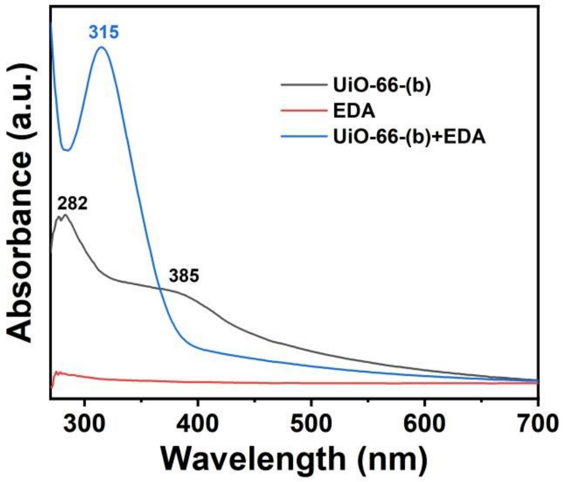

Fig. S14 UV-vis spectra of UiO-66-(b) before and after contact with ethylenediamine (EDA).

The UV-vis spectra of UiO-66-(b) before and after contact with EDA were compared. From **Fig. S14**, it can be seen that UiO-66- (b) itself possesses absorption peaks at 282 nm and 385 nm. After contact with EDA, the absorption peak at 385 nm disappears, while a new absorption peak appears at 315 nm, indicating the formation of new bonds or groups and the formation of new substances. This also proves the successful coupling between UiO-66-(b) and amines.

Table S1 Comparison with other methods for formaldehyde detection

Materials	Methods	Linear range /μM	LOD /μM	Reference
Fluorescent probe NaP	Fluorescence	0~10	1.62	1
PET-based fluorescent probe	Fluorescence	2~10	1.5	2
HBQP-capped quantum dots (QDs)	Fluorescence	1~28	0.49	3
Tb^{3+}-doped Ag-MOFs	Fluorescence	100~1000	1.9	4
UiO-66-HN	Fluorescence	20~60	0.167	5
Carbon dots (B-CDs)	Fluorescence	0.67~33	0.25	6
Quinolimide-based fluorescent probe	Fluorescence	0~180	1.7	7
R6G@UiO-66-(a)	Fluorescence	0.2~6.8	0.058	This work

Reference:

1. Xin F, Tian Y, Jing J, et al. A two-photon fluorescent probe for imaging of endogenous formaldehyde in HeLa cells and quantitative detection of basal formaldehyde in milk samples. *Analytical Methods*, 2019, 11(23): 2969-2975.

2. Cheng H, Zou L, Yang L, et al. A turn-on fluorescence probe for rapid, sensitive and visual detection of formaldehyde. *ChemistrySelect*, 2019, 4(1): 432-436.

3. Ahmad I, Zhou Z, Li H Y, et al. Crafting CdTe/CdS QDs surface for the selective recognition of formaldehyde gas via ratiometric contrivance. *Sensors and Actuators B: Chemical*, 2020, 304: 127379.

4. Li M, Shen A, Du M, et al. Tb^{3+}-Doped Ag-MOFs for fluorescent detection of formaldehyde in a novel smartphone platform and its removal applications in milk products and wastewater. *RSC Advances*, 2021, 11(54): 34291-34299.

5. Li X, Qu H, Wang Y, et al. Fluorescent probe for detection of formaldehyde based on UiO-66-NH_2. *Journal of Solid State Chemistry*, 2023, 317: 123672.

6. Li Y, Hu M, Liu K, et al. Lignin derived multicolor carbon dots for visual detection of formaldehyde. *Industrial Crops and Products*, 2023, 192: 116006.

7. Cao H, Yang J, Zhang Y, et al. A simple quinolimide-based fluorescent sensor for formaldehyde and its applications in test strips and living cells. *Journal of Photochemistry and Photobiology A: Chemistry*, 2023, 444: 115023.

Table S2 Comparison with other methods for ethylenediamine detection

Materials	Methods	Linear range /μM	LOD /μM	Reference
PEBBO-based LB film	Fluorescence	5.5~136	0.27	1
Tb^{3+}-metal–organic gels (MOGs)	Fluorescence	10~200	3.8	2
Zn(II) coordination polymer	Fluorescence	0~400	0.065	3
Benzothiadiazole-based compound	Fluorescence	0~7.4	0.635	4
Water-soluble pillar[4]arene[1]quinone	Fluorescence	20~80	0.96	5
Xanthene-based fluorescent probe	Fluorescence	0~10	0.054	6
Ag^{+}@Zn-MOFs	Fluorescence	10~135	0.027	7
R6G@UiO-66-(b)	Fluorescence	0.005~0.275	0.0017	This work

Reference:

1. Shang C, Wang L, An Y, et al. Langmuir–Blodgett films of perylene bisimide derivatives and fluorescent recognition of diamines. *Physical Chemistry Chemical Physics*, 2017, 19(35): 23898-23904.

2. Gu D, Yang W, Lin D, et al. Water-stable lanthanide-based metal–organic gel for the detection of organic amines and white-light emission. *Journal of Materials Chemistry C*, 2020, 8(39): 13648-13654.

3. Ma J, Zhou T, Ma T, et al. Construction of transition metal coordination polymers with free carboxyl groups and turn-on fluorescent detection for α, β-diamine. *Crystal Growth & Design*, 2020, 21(1): 383-395.

4. Qiu C Q, Li L Q, Yao S L, et al. Two benzothiadiazole-based compounds as multifunctional fluorescent sensors for detection of organic amines and anions. *Polyhedron*, 2021, 199: 115100.

5. Wang J, Cen M, Wang J, et al. Water-soluble pillar [4] arene [1] quinone: Synthesis, host-guest property and application in the fluorescence turn-on sensing of ethylenediamine in aqueous solution, organic solvent and air. *Chinese Chemical Letters*, 2022, 33(3): 1475-1478.

6. Priya B, Kumar N. Reaction-based fluorescent detection of diamines via tuning the probe aggregation. *Journal of Photochemistry and Photobiology A: Chemistry*, 2023, 445: 115036.

7. Liu L, Chen X L, Cai M, et al. Zn-MOFs composites loaded with silver nanoparticles are used for fluorescence sensing pesticides, Trp, EDA and photocatalytic degradation of organic dyes. *Spectrochimica Acta Part A: Molecular and Biomolecular Spectroscopy*, 2023, 289: 122228.
